# Integrated Transcriptomics and Metabolomics with Machine Learning Identify Flavonoids as Key Effectors in Wheat Root Thermotolerance

**DOI:** 10.3390/plants15060965

**Published:** 2026-03-20

**Authors:** Wenyuan Shen, Qingming Ren, Yiyang Dai, Yu Zhang, Fei Xiong

**Affiliations:** College of Bioscience and Biotechnology, Yangzhou University, Yangzhou 225009, China; swy119611@126.com (W.S.); dx120230212@stu.yzu.edu.cn (Q.R.);

**Keywords:** high temperature, wheat root, transcriptome, metabolome, machine learning

## Abstract

Root plasticity is vital for crop survival amid global warming. Yet, the molecular mechanisms governing wheat root thermotolerance remain largely unknown. In this study, we combined phenomics, transcriptomics, and metabolomics with machine learning to analyze the performance of heat-tolerant cultivar YM158 and heat-sensitive cultivar YM15 under varying heat stress. While high temperatures (35 °C) severely inhibited root growth and caused oxidative damage in YM15, YM158 maintained robust root architecture and redox balance. Using weighted gene co-expression network analysis (WGCNA) alongside the random forest feature selection algorithm, we identified the flavonoid biosynthesis pathway as central to thermotolerance. Protein–protein interaction network analysis revealed that wheat root adaptability to high temperatures involves maintaining protein homeostasis via the endoplasmic reticulum protein processing system, specifically activating the flavonoid biosynthesis pathway and enhancing the antioxidant enzyme system. Furthermore, we identified a potential regulatory hub involving the cell wall sensor FERONIA (FER) and heat shock factors (HSFs), highlighting a complex interaction between hormonal signaling and secondary metabolism. Our study offers a detailed map of root heat adaptation and positions the flavonoid-mediated antioxidant system as a promising target for breeding climate-resilient crops.

## 1. Introduction

Global warming poses a significant threat to global food security [1]. Predictions suggest that extreme high-temperature events will become more frequent and intense in the coming decades [2]. Wheat (*Triticum aestivum* L.), a staple crop supplying calories and proteins to about 20% of the global population, is particularly vulnerable to heat stress [3]. High temperatures during the wheat growth cycle hinder photosynthesis and grain filling, cause oxidative cell damage, and lead to metabolic disorders, resulting in considerable yield losses and quality decline [4,5]. While the heat tolerance of aboveground parts has been extensively studied, the adaptability of roots, crucial for sensing soil temperature changes, is often neglected. Roots are essential for water and nutrient absorption and are primary sites for synthesizing various hormones and secondary metabolites [6,7]. Due to soil’s limited temperature buffering capacity, roots endure prolonged heat stress compared to aboveground parts [8]. Thus, understanding the molecular regulatory network of wheat roots in response to high temperatures is strategically vital for developing climate-resilient crops.

Plant adaptation to high temperatures is a complex process involving the integration of multiple signals, with plant hormones playing a crucial regulatory role [9]. The hormone network not only transmits long-distance systemic signals but also finely regulates the Growth-Defense Trade-off [10]. Abscisic acid, a classic stress hormone, rapidly accumulates under high temperatures, inducing the expression of stress-resistance genes and regulating root water status [11]. Ethylene’s role in high temperatures is more complex; it remodels root system architecture by regulating root cell-wall extensibility but may also accelerate root senescence [12]. Recently, the roles of auxin and cytokinin in heat tolerance have gained attention. High temperatures often disrupt the auxin gradient, leading to root growth arrest [13]. Heat-tolerant plants respond to high temperatures through a finely tuned hormone interaction network [14]. Recent studies further emphasize that these phytohormone-mediated responses are governed by dynamic gene regulatory networks that link developmental processes with environmental adaptation [15]. Moreover, master transcription factors from several regulatory families have been identified as key integrators of multiple hormone pathways during stress priming and adaptive responses [16]. For instance, ABA-dependent activation of defense pathways and stress-responsive genes plays a central role in amplifying these hormonal signals for optimal plant stress adaptation [17]. However, the mechanisms by which roots sense high-temperature signals, convert them into intracellular hormone cascade reactions, and activate the downstream metabolic defense system’s regulatory network remain to be further elucidated.

In the intricate defense mechanisms of plants, secondary metabolites, particularly flavonoids from the phenylpropanoid pathway, are emerging as a focal point in heat-tolerance research [18]. Traditionally, flavonoids have been viewed primarily as non-enzymatic antioxidants [19]. Their structures, rich in phenolic hydroxyl groups, enable them to effectively capture and neutralize reactive oxygen species, often outperforming enzymatic antioxidant systems like superoxide dismutase (SOD) and catalase (CAT). This is especially crucial when extreme heat inactivates enzyme proteins, positioning flavonoids as the final antioxidant defense in cells [20]. However, growing evidence suggests that flavonoids have roles beyond this. Compounds such as naringenin and quercetin can modulate the polar transport of auxin by interacting with certain protein kinases or transporters, influencing root gravitropic responses and lateral root development [21,22,23]. This dual regulatory mechanism of metabolite-hormone implies that flavonoids might be pivotal in linking metabolic adaptation with morphological changes. Although transcriptomic studies have consistently shown the activation of flavonoid synthesis genes under high temperatures, there is a notable lack of comprehensive multi-omics evidence detailing the specific metabolic accumulation of key flavonoid components, like naringenin and luteolin, in heat-tolerant germplasms, as well as the molecular mechanisms through which they synergistically mediate root heat tolerance with hormonal signals.

With the rapid advancement of bioinformatics tools and resources, integrating multi-omics data has become a common and efficient method for analyzing complex response processes under environmental stress [24]. The transcriptome reveals potential gene expression, while the metabolome reflects the ultimate physiological and biochemical phenotypes [25]. Combining these two data types with advanced data mining strategies is essential for accurately identifying key regulatory pathways [26]. Traditional statistical methods struggle with high-dimensional omics data, but recent machine learning algorithms, such as Random Forests, offer powerful tools for screening driver biomarkers from vast datasets. These algorithms enable us to more sensitively detect the key metabolic features crucial for heat tolerance [27].

In this study, we examined two wheat cultivars (heat-sensitive YM15 and heat-tolerant YM158) that exhibit notable differences in heat tolerance in the field. By simulating temperature stresses across various gradients, we systematically analyzed the phenotypic, physiological, transcriptomic, and metabolomic responses of their roots. Our goal was to elucidate the specific advantages of heat-tolerant cultivars in maintaining root architecture and redox homeostasis. Through a multi-omics joint analysis, we aimed to precisely identify the key metabolic pathways and regulatory networks that determine heat tolerance, thereby uncovering potential mechanisms related to heat tolerance in wheat roots. Our findings revealed the dual functions of the flavonoid biosynthesis pathway in enhancing heat tolerance in wheat roots, offering a crucial theoretical basis and genetic resources for breeding new wheat cultivars with robust roots, heat tolerance, and stable yields.

## 2. Results

### 2.1. Phenotypic and Physiological Responses of Wheat Roots Under High-Temperature Stress

To assess the impact of high-temperature stress on the root growth of various wheat cultivars, we examined the morphological and physiological indicators of a heat-sensitive cultivar (YM15) and a heat-tolerant cultivar (YM158) under temperature treatments (25 °C, 30 °C, and 35 °C).

**Figure 1:**
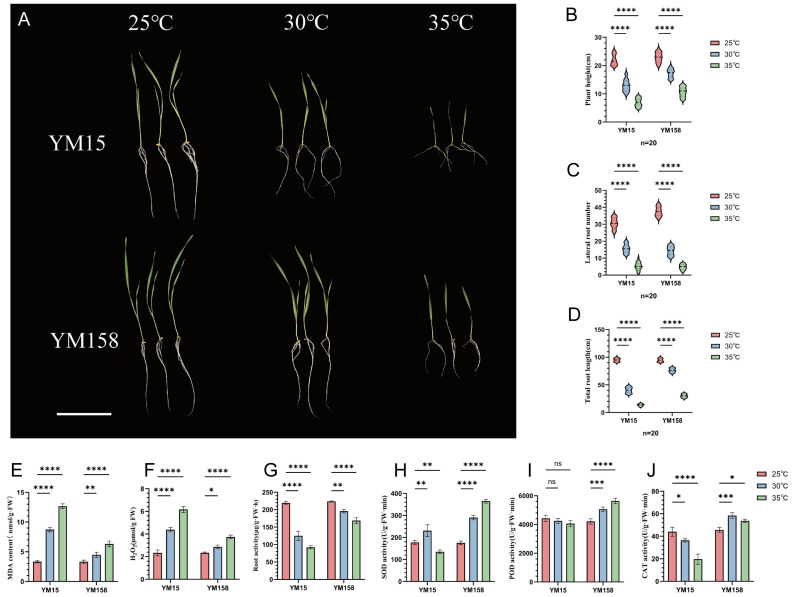
Phenotypic and physiological characterization of heat-tolerant (YM158) and heat-sensitive (YM15) wheat cultivars under graded temperature stress. (**A**) Representative photographs showing plant morphology of YM15 and YM158 treated at 25 °C, 30 °C, and 35 °C for 5 days. Scale bar = 10 cm. (**B**) Violin plot of plant height. (**C**) Violin plot of the number of lateral roots. (**D**) Violin plot of total root length. (**E**) Malondialdehyde (MDA) content in roots. (**F**) Hydrogen peroxide (H_2_O_2_) content in roots. (**G**) Root activity measured by TTC reduction method. (**H**) Superoxide dismutase (SOD) activity in roots. (**I**) Peroxidase (POD) activity in roots. (**J**) Catalase (CAT) activity in roots. For violin plots (**B**–**D**), the width of the shaded area represents the probability density of the data, and the internal box plots indicate the median (central dot) and interquartile range (n = 20 biological replicates). For physiological indices (**E**–**J**), data represent means ± SD (n = 3 biological replicates). Asterisks indicate statistically significant differences between the compared groups based on one-way ANOVA (* *p* < 0.05, ** *p* < 0.01, *** *p* < 0.001, **** *p* < 0.0001; ns, not significant).

High-temperature stress markedly inhibited wheat growth. As temperatures rose, both cultivars exhibited declines in total root length, number of lateral roots, and plant height (Figure 1A). Nonetheless, YM158 demonstrated greater heat tolerance compared to YM15. Under extreme high-temperature stress at 35 °C, YM15’s total root length decreased significantly by 85.7% relative to the control at 25 °C, whereas YM158 experienced a smaller reduction of 68.3% (Figure 1D). Furthermore, the decrease in plant height for YM158 under high temperatures was 53.6%, significantly less than the 68.4% decrease observed in YM15, suggesting that YM158 maintains relatively better growth under heat stress (Figure 1B). Additionally, lateral root development was significantly inhibited in both cultivars (Figure 1C).

As temperatures rise, the reactive oxygen species (ROS) burst and membrane lipid peroxidation in the heat-sensitive cultivar YM15 become extremely severe. At an extreme temperature (35 °C), the H_2_O_2_ content in YM15 increased sharply by 164.3% compared to the control at 25 °C, while malondialdehyde (MDA) content increased substantially by 279.1%, indicating irreversible and severe damage to its cell membrane system (Figure 1F). In contrast, at 35 °C, the increases in H_2_O_2_ and MDA contents in YM158 were only 59.7% and 89.3%, respectively, significantly lower than those in YM15. This suggests that YM158 more effectively scavenges excess ROS and maintains cell membrane integrity (Figure 1E). High temperatures significantly inhibit the metabolic activity of wheat roots. After exposure to 30 °C and 35 °C, YM15 root activity decreased by 43.0% and 58.0%, respectively, indicating a rapid decline in physiological functions. YM158 maintained relatively high activity under high temperatures, with a decline of only 24.4% at 35 °C, about half that of YM15 (Figure 1G). These results confirm that YM158 sustains strong respiratory metabolism and absorption capacity under high-temperature stress, providing a physiological basis for its heat tolerance.

To investigate the physiological mechanisms behind the phenotypic differences, we measured the activities of the root antioxidant enzyme system. The results revealed distinct regulatory patterns for antioxidant enzymes between the two cultivars. In YM158, the activities of SOD, POD, and CAT increased significantly with rising temperatures. Notably, under the 35 °C treatment, SOD and POD activities in YM158 were 108.2% and 33.9% higher than those in the control, respectively, indicating a rapid activation of its antioxidant system to scavenge reactive oxygen species (Figure 1H,I). Conversely, the antioxidant system in YM15 was severely impaired at high temperatures. At 35 °C, the activities of SOD, POD, and CAT in YM15 were all lower than in the control, with CAT activity decreasing by up to 55.3% (Figure 1J). These findings suggest that YM158 mitigates oxidative damage by maintaining high levels of antioxidant enzyme activities, thereby demonstrating stronger heat tolerance.

### 2.2. Metabolomic Analysis

#### 2.2.1. Metabolic Reprogramming of Wheat Roots in Response to High-Temperature Stress

**Figure 2:**
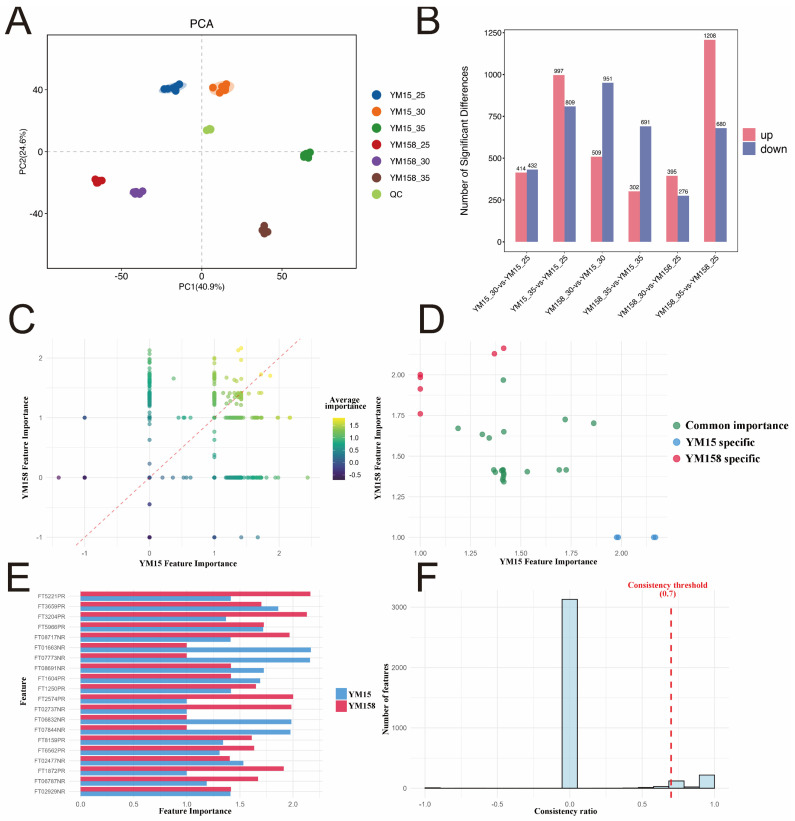
Metabolomic profiling and machine learning-based screening of key metabolic biomarkers in wheat roots under heat stress. (**A**) Principal component analysis (PCA) score plot of the metabolome for YM15 and YM158 under 25 °C, 30 °C, and 35 °C, showing the overall separation of samples. (**B**) Statistical summary of differentially accumulated metabolites (DAMs) in different comparison groups. (**C**) Correlation analysis of feature importance scores between YM15 and YM158 derived from the Random Forest model. The red dashed line represents the 1:1 reference line ($y = x$), indicating equal feature importance between the two cultivars. (**D**) Visualization of sample classification based on the selected metabolic features (e.g., t-SNE or clustering heatmap). (**E**) Comparison of the Top 20 metabolic features ranked by their importance scores. (**F**) Distribution of feature importance consistency, indicating the stability of the selected biomarkers across the two cultivars. PCA was performed based on all detected metabolic features. Feature importance scores were calculated using the Random Forest algorithm. DAMs were identified based on VIP > 1 and *p* < 0.05. All metabolomic data represent six independent biological replicates (n = 6) per treatment group.

To investigate how wheat responds to high temperatures at the metabolic level, we utilized non-targeted metabolomics to analyze the root metabolic profiles of a heat-sensitive cultivar (YM15) and a heat-tolerant cultivar (YM158) under various temperature conditions. Principal component analysis (PCA) results revealed that PC1 and PC2 explained 40.9% and 24.6% of the total variance, respectively (Figure 2A). The samples clustered distinctly according to temperature in the PCA space, demonstrating that high temperatures induce significant metabolic reprogramming.

Further analysis of differential accumulated metabolites (DAMs) revealed that the intensity of metabolic responses to high temperatures in the two cultivars significantly increased as stress intensified (Figure 2B). Under moderate high-temperature stress (30 °C vs. 25 °C), 846 DAMs were identified in YM15, with 414 up-regulated and 432 downregulated. In contrast, only 671 DAMs were detected in YM158, comprising 395 up-regulated and 276 downregulated. This suggests that at 30 °C, the metabolic profile of the heat-tolerant cultivar YM158 remained relatively more stable and less disrupted.

When the temperature rose to an extreme 35 °C, the metabolic responses of the two cultivars diverged significantly. In YM15, the total number of differentially accumulated metabolites (DAMs) increased to 1806, with 997 up-regulated and 809 down-regulated. In contrast, YM158 demonstrated a stronger metabolic mobilization, identifying a total of 1888 DAMs. Notably, at 35 °C, YM158 had 1208 up-regulated metabolites, significantly more than YM15, while the down-regulated metabolites numbered only 680. This shift from relative homeostasis at 30 °C to substantial up-regulation at 35 °C may enable YM158 to rapidly establish a new metabolic balance under extreme high temperatures.

#### 2.2.2. Identification of Key Response Features Based on Machine Learning

Traditional statistical methods struggle with high-dimensional omics data, but recent machine learning algorithms, such as Random Forests, offer powerful tools for screening driver biomarkers from vast datasets. Machine learning addresses the limitations of traditional differential analysis, which focuses solely on fold change, by identifying features with small fold changes that play central roles in regulatory networks. To accurately pinpoint the key metabolic features that determine variations in heat tolerance, we employed a Random Forest-based feature selection algorithm. Specifically, we developed classification models where the temperature treatment (25 °C, 30 °C, 35 °C) was the target variable, and the 3546 identified metabolic features served as predictor variables. By independently constructing RF classifiers configured with 1000 decision trees for YM15 and YM158, we utilized the Mean Decrease Accuracy (MDA) to assess the significance and predictive contribution of each metabolite. This approach allowed for a more robust integrated analysis of the metabolic features (Figure 2C–F). Spearman correlation analysis revealed a correlation coefficient of just 0.106 (*p* < 0.001) for the feature importance rankings between YM15 and YM158. This notably low correlation indicates that the two cultivars employ different metabolic regulation strategies to cope with high-temperature stress.

We conducted a feature importance and consistency analysis, identifying 359 features with high consistency, 726 features favored by YM15, and 690 features preferred by YM158. From these, we pinpointed 30 key metabolites that significantly contributed to the model (Figure 2C,E). Of these metabolites, 20 were common important features, exhibiting high importance in both cultivars, with an average importance score of 1.48 and a consistency of 0.909, reflecting the core metabolic pathways in wheat’s response to high temperatures. Notably, among the 30 key metabolites, 4 were specific to YM15 and 6 were specific to YM158 (Figure 2D). The 6 metabolites with high predictive contributions in the heat-tolerant cultivar YM158 (average importance score > 1.5) are likely crucial drivers of its heat tolerance.

#### 2.2.3. Screening of Key Biomarkers for Heat Tolerance

**Figure 3:**
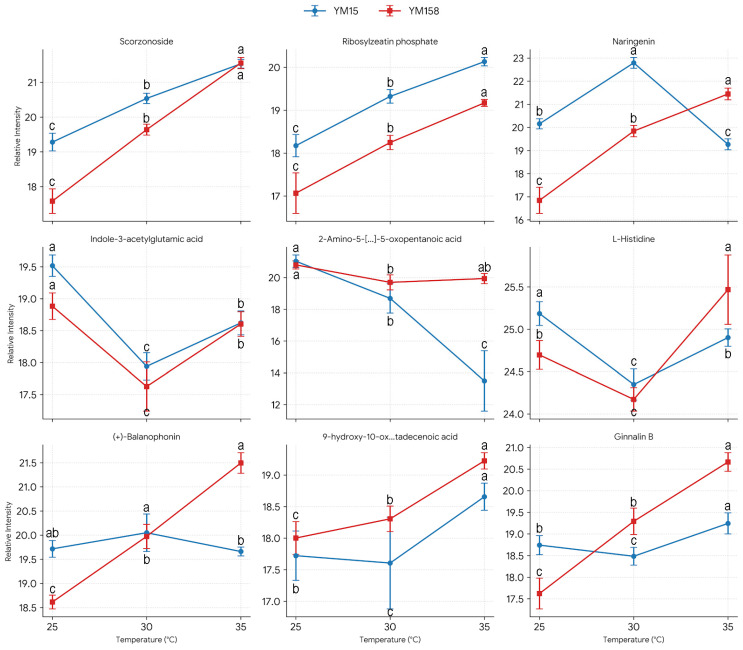
Accumulation patterns of the nine key metabolic biomarkers screened by machine learning in wheat roots under heat stress. Relative abundance of the top 9 metabolic features prioritized by the machine learning model in YM15 and YM158 under 25 °C, 30 °C, and 35 °C treatment. Data represent means ± SD (n = 6 biological replicates). Different lowercase letters indicate statistically significant differences between treatments and cultivars at *p* < 0.05 (one-way ANOVA followed by Duncan’s multiple range test). 2-Amino-5-[…]-5-oxopentanoic acid: 2-Amino-5-[[1-[carboxymethyl(sulfo)amino]-1-oxo-3-sulfanylpropan-2-yl]amino]-5-oxopentanoic acid. 9-hydroxy-10-ox…tadecenoic acid: 9-hydroxy-10-oxo-12Z-octadecenoic acid.

To pinpoint the core effector molecules responsible for heat tolerance in YM158 from extensive metabolic data, we selected the top 100 metabolic features using the Feature Importance Score from a machine learning model. We then conducted an expression level analysis to further filter metabolites that were either highly expressed or significantly up-regulated in the heat-tolerant cultivar YM158. Ultimately, we identified 9 key biomarkers (Figure 3) that primarily fall into three categories: hormone regulation, secondary metabolic defense, and signal transduction.

We identified hormonal homeostasis regulators, such as ribosylzeatin phosphate, a precursor of cytokinin, and indole-3-acetylglutamic acid (IAA conjugate). The accumulation of ribosylzeatin phosphate helps delay root senescence induced by high temperatures. Meanwhile, IAA-Glu acts as an auxin reservoir, potentially aiding YM158 in maintaining auxin homeostasis during stress.

Naringenin, ginnalin B, (+)-Balanophonin, and scorzonoside were significantly enriched in YM158. Naringenin serves as a key intermediate in flavonoid biosynthesis and exhibits strong free radical scavenging properties. Ginnalin B, a type of gallotannin, possesses antioxidant activity, while Balanophonin, a lignan, may contribute to cell wall reinforcement. The accumulation of these secondary metabolites creates a chemical defense barrier for YM158, directly confirming the activation of the Phenylpropanoid biosynthesis pathway.

We identified the oxidized lipid 9-hydroxy-10-oxo-12Z-octadecenoic acid, which typically functions as a signaling molecule involved in stress early warning, similar to jasmonic acid precursors. Additionally, the high levels of L-Histidine and the sulfur-containing amino acid derivative 2-Amino-5-[[1-[carboxymethyl(sulfo)amino]-1-oxo-3-sulfanylpropan-2-yl]amino]-5-oxopentanoic acid indicate that YM158 may mitigate high-temperature toxicity by either chelating metal ions or regulating sulfur metabolism, such as in glutathione-related pathways.

### 2.3. Transcriptome Analysis

#### 2.3.1. Transcriptional Reprogramming of Wheat Roots in Response to High-Temperature Stress

**Figure 4:**
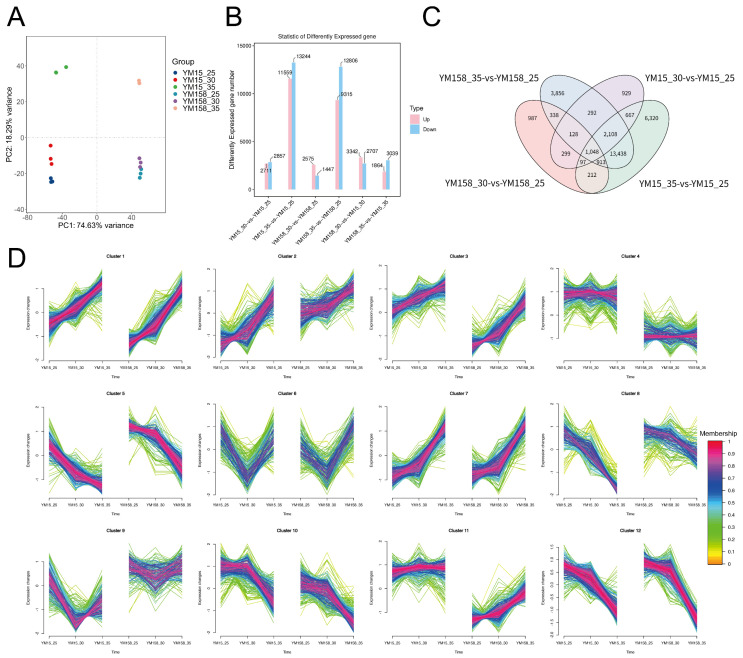
Transcriptomic landscape and expression pattern analysis of wheat roots under graded temperature stress. (**A**) Principal component analysis (PCA) score plot based on the global gene expression profiles of YM15 and YM158 under 25 °C, 30 °C, and 35 °C. (**B**) Statistical summary of the number of up- and down-regulated differentially expressed genes (DEGs) in different comparison groups. (**C**) Venn diagram illustrating the unique and overlapping DEGs among comparison groups. (**D**) K-means clustering analysis of DEGs, grouping genes into distinct clusters based on their expression trends across treatments.

To thoroughly examine gene expression patterns in wheat roots subjected to high-temperature stress, we conducted transcriptome sequencing (RNA-seq) on YM15 and YM158. Principal component analysis (PCA) showed that PC1 and PC2 explained 74.63% and 18.29% of the total variance, respectively (Figure 4A). The high explanatory power of PC1 suggested that temperature was the primary factor influencing transcriptome variation, with distinct separation of treatment groups in the PCA space.

The statistical analysis of differentially expressed genes (DEGs) revealed significant changes in gene expression for both cultivars as temperature increased (Figure 4B). Under moderate stress (30 °C vs. 25 °C), the heat-sensitive cultivar YM15 showed more pronounced transcriptional fluctuations, with 5568 DEGs (2711 up-regulated and 2857 down-regulated). In contrast, the heat-tolerant cultivar YM158 experienced relatively smaller changes, with 4022 DEGs. Notably, the number of down-regulated genes in YM158 (1447) was only about half of that in YM15 (2857), indicating YM158’s stronger ability to maintain transcriptional homeostasis under moderate high temperatures. Under extreme stress (35 °C vs. 25 °C), the total number of DEGs in YM15 and YM158 increased dramatically to 24,803 and 22,121, respectively. In YM158, 9315 genes were up-regulated while 12,806 were down-regulated. Although both cultivars exhibited similar magnitudes of change, this genome-wide reprogramming underscores the complex regulatory network of wheat in response to lethal temperatures.

#### 2.3.2. Clustering of Core Responsive Genes and Expression Patterns

To differentiate between the common response mechanisms and specific heat tolerance mechanisms of the two cultivars, we employed Venn diagrams to examine the overlaps of differentially expressed genes (DEGs) (Figure 4C). The analysis revealed that 1048 genes were significantly altered across all high-temperature treatment groups, representing the conserved core transcriptome of wheat in response to high-temperature stress. More notably, under the extreme high temperature of 35 °C, 3856 genes were specifically induced in the heat-tolerant cultivar YM158. These genes did not show significant changes or exhibited different trends in the heat-sensitive cultivar YM15. This indicates that YM158 possesses a unique transcriptional regulatory branch to withstand lethal high temperatures.

To better understand the spatiotemporal dynamics of gene expression, we used the K-means clustering algorithm to categorize all differentially expressed genes (DEGs) into 12 primary expression patterns (Figure 4D). By examining the expression trends of each cluster across different cultivars and temperatures, we pinpointed Cluster 2, Cluster 8, and Cluster 9 as key candidates responsive to high temperatures. These clusters showed notably high expression or specific upregulation in the high-temperature treatment group (particularly at 35 °C) of the heat-tolerant cultivar YM158, while their expression levels were low or suppressed in the heat-sensitive cultivar YM15. This pattern suggests that Clusters 2, 8, and 9 may harbor crucial regulatory factors and functional genes that confer heat tolerance to YM158.

#### 2.3.3. Functional Enrichment Analysis

To systematically uncover the molecular mechanisms underlying wheat’s response to high temperatures and to decipher the adaptation strategies of the heat-tolerant cultivar YM158, we performed GO and KEGG enrichment analyses on the differentially expressed genes.

**Figure 5:**
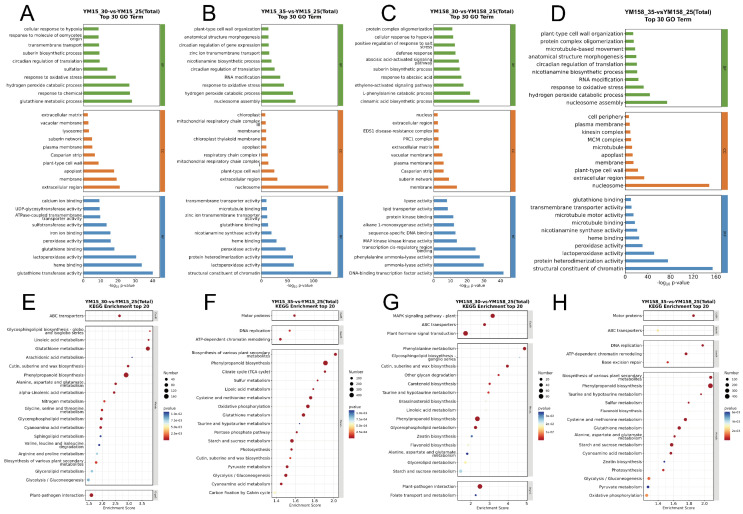
Functional annotation and pathway enrichment analysis of differentially expressed genes (DEGs) in heat-sensitive (YM15) and heat-tolerant (YM158) wheat cultivars. (**A**) Gene Ontology (GO) enrichment analysis of DEGs in YM15 (30 °C vs. 25 °C). (**B**) GO enrichment analysis of DEGs in YM15 (35 °C vs. 25 °C). (**C**) GO enrichment analysis of DEGs in YM158 (30 °C vs. 25 °C). (**D**) GO enrichment analysis of DEGs in YM158 (35 °C vs. 25 °C). (**E**) Kyoto Encyclopedia of Genes and Genomes (KEGG) pathway enrichment analysis of DEGs in YM15 (30 °C vs. 25 °C). (**F**) KEGG pathway enrichment analysis of DEGs in YM15 (35 °C vs. 25 °C). (**G**) KEGG pathway enrichment analysis of DEGs in YM158 (30 °C vs. 25 °C). (**H**) KEGG pathway enrichment analysis of DEGs in YM158 (35 °C vs. 25 °C). The top 30 most significantly enriched GO terms and top 20 most significantly enriched KEGG pathways are displayed. The size of the dots represents the number of DEGs mapped to the term/pathway, and the color scale indicates the significance level (*p*-value).

GO annotation analysis (Figure 5A–D) highlighted notable differences in biological processes between the two cultivars under varying heat intensities. At the extreme temperature of 35 °C, both cultivars adopted similar basic survival strategies. The significantly enriched terms shared by both included response to oxidative stress, hydrogen peroxide catabolic process, and nucleosome assembly. This suggests that at this lethal temperature, the primary functions of wheat roots are to eliminate excess reactive oxygen species (ROS) and restructure chromatin to maintain genomic stability (Figure 5B,D). At the moderate temperature of 30 °C, the heat-tolerant cultivar YM158 exhibited unique signal and metabolic regulation processes. Specifically enriched in the ethylene-activated signaling pathway and cinnamic acid biosynthetic process, YM158 appeared to initiate hormone signaling and secondary metabolic barriers early in the stress response. In contrast, the sensitive cultivar YM15 was enriched only in the glutathione metabolic process, indicating its response was largely confined to simple antioxidant biochemical reactions, lacking extensive signal transduction regulation (Figure 5A,C).

KEGG pathway analysis (Figure 5E–H) further elucidated the key molecular networks underlying the aforementioned biological processes. At 30 °C, YM158 demonstrated a robust ability to perceive signals, with its up-regulated genes significantly enriched in the MAPK Signaling Pathway—Plant and Plant Hormone Signal Transduction. This suggests that YM158 can swiftly convert external temperature signals into intracellular transcriptional signals via the MAPK cascade and hormone signaling networks. In contrast, YM15 was primarily enriched in Glutathione Metabolism at this stage, indicating a lack of an active signal regulatory network and a reliance on passive defense mechanisms (Figure 5E,G). When the temperature increased to 35 °C, the differences in metabolic pathways between the two determined their ultimate heat tolerance. YM158 activated the Protein Processing in Endoplasmic Reticulum pathway, which is typically associated with protein repair capabilities and showed highly significant enrichment (Figure 5F,H). Conversely, although pathways related to antioxidant response (Glutathione Metabolism) and photosynthesis (Photosynthesis) were significantly enriched in YM15, there was no significant enrichment detected for the Protein Processing in Endoplasmic Reticulum pathway.

### 2.4. WGCNA

#### 2.4.1. Construction of Gene-Metabolite Co-Expression Network and Identification of Key Modules

**Figure 6:**
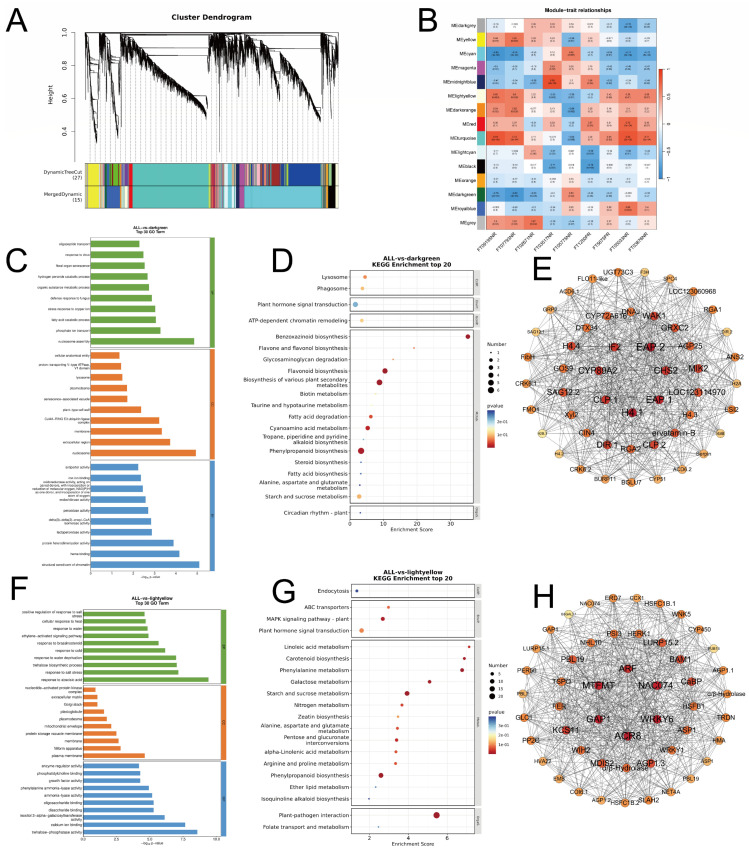
Weighted gene co-expression network analysis (WGCNA) identifies key regulatory modules associated with metabolic adaptations in wheat roots. To integrate the transcriptomic and metabolomic datasets, the relative abundances of the 9 key metabolic biomarkers (identified via machine learning) were utilized as phenotypic trait data and correlated against the transcriptomic gene co-expression modules. (**A**) Hierarchical clustering dendrogram of co-expressed genes. The color branches represent distinct modules identified by dynamic tree cutting. The color branches and the horizontal color bands beneath the tree indicate the distinct modules identified by dynamic tree cutting and merging. Each color serves as an arbitrary identifier for a specific co-expression module containing genes with highly correlated expression profiles, whereas the grey color represents background genes that could not be assigned to any distinct module. (**B**) Module-trait relationship heatmap illustrating the correlation between module eigengenes and the accumulation of key metabolic biomarkers. The relationships between gene expression and metabolite accumulation were statistically evaluated using Pearson correlation analysis. The color scale represents the Pearson correlation coefficient, with red indicating positive correlation and blue indicating negative correlation. The exact correlation coefficient and its corresponding statistical significance (*p*-value) are displayed directly within each cell of the heatmap. (**C**–**E**) Functional characterization of the dark-green module: GO enrichment analysis (**C**), KEGG pathway enrichment analysis (**D**), and Protein–Protein Interaction (PPI) network of the top hub genes (**E**). (**F**–**H**) Functional characterization of the light-yellow module: GO enrichment analysis (**F**), KEGG pathway enrichment analysis (**G**), and PPI network of the top hub genes (**H**). In the PPI networks (**E**,**H**) (Tables S3 and S4), node size and color intensity are directly proportional to the degree of connectivity (number of interacting edges), and edge thickness indicates the strength of the interaction (confidence score).

To develop a systematic regulatory map linking metabolic markers with gene networks, we constructed an integrated weighted gene co-expression network (WGCNA). This network utilized transcriptomic data to analyze nine selected key metabolites as target traits. By applying a soft threshold and building a hierarchical clustering tree, we grouped the differentially expressed genes into 14 co-expression modules (Figure 6A). Notably, the dark-green and light-yellow modules exhibited the strongest correlations with the key metabolites specifically accumulated in the heat-tolerant cultivar YM158, making them the focus of our in-depth analysis (Figure 6B).

The light-yellow module demonstrates exceptionally high correlations with various hormones and signal-related metabolites. Its correlation coefficients with ribosylzeatin phosphate (*r* = 0.6, *p* = 0.008) and indole-3-acetylglutamic acid (*r* = −0.63, *p* = 0.005) are both statistically significant. Meanwhile, the dark-green module exhibits a strong co-expression pattern with secondary metabolites and antioxidant molecules, showing significant correlations with naringenin (*r* = −0.63, *p* = 0.005) and scorzonoside (*r* = −0.76, *p* = 3 × 10^−4^).

#### 2.4.2. Enrichment Analysis of Key Module Functions and Protein–Protein Interaction Network Analysis

To unravel the molecular mechanisms by which the dark-green and light-yellow modules regulate heat tolerance, we performed functional enrichment and protein–protein interaction (PPI) network analyses.

The dark-green module is closely linked to the synthesis of secondary metabolites and basic defense responses. KEGG pathway analysis (Figure 6D) showed significant enrichment in Flavonoid biosynthesis, Phenylpropanoid biosynthesis, and Plant hormone signal transduction. GO functional annotation (Figure 6C) further highlighted significant enrichment in processes like Hydrogen peroxide catabolic process, Peroxidase activity, and Nucleosome assembly. PPI network analysis (Figure 6E) identified the Top 50 core hub genes with the highest connectivity within this module. Among the metabolic genes, key enzymes in the flavonoid synthesis pathway were identified, including chalcone synthase 2 (CHS) and naringenin 2-oxoglutarate 3-dioxygenase. In the antioxidant enzyme category, Cationic peroxidase *SPC4* emerged as a core hub gene. The network also included multiple histone variants, such as Histone H4 variant TH011 and Histone H2B.3.

Unlike the dark-green module, which emphasizes metabolic execution, the light-yellow module displays typical signal-transcription regulatory features. KEGG analysis (Figure 6G) revealed significant enrichment of this module in the MAPK Signaling Pathway—Plant and Plant Hormone Signal Transduction. GO annotation indicated enrichment in various hormone response processes (Figure 6F), such as the Response to abscisic acid (ABA response), Ethylene-activated signaling pathway, and Response to brassinosteroid. PPI network analysis (Figure 6H) identified several hub genes involved in signal transduction and transcriptional regulation. Among the transcription factors were Heat stress Transcription Factor B-1 (HSF B-1), HSF C-1b, WRKY transcription factor 6, and NAC domain-containing protein 74. In terms of receptor kinases, Receptor-like protein kinase FER and Leucine-rich repeat receptor-like serine/threonine-protein kinase BAM1 were identified. Additionally, the ABA-induced proteins, Early-Responsive to Dehydration 7 (ERD7) and Protein HVA22, were central to the network.

### 2.5. Important Pathways of Root System Response to High-Temperature Stress

**Figure 7:**
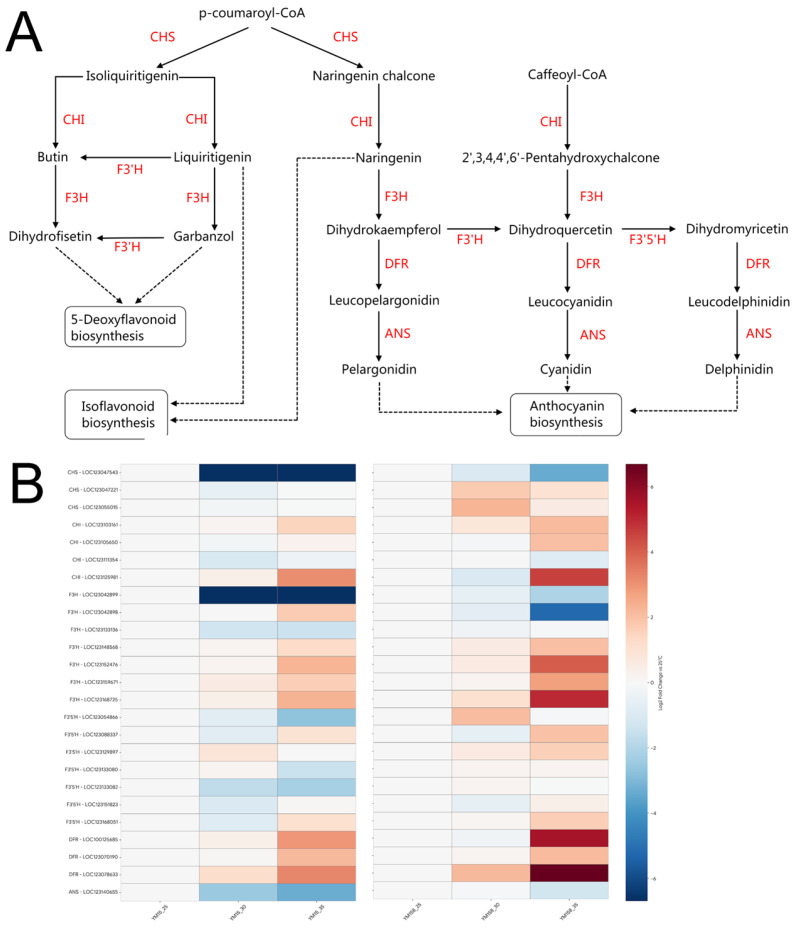
Schematic representation of the flavonoid biosynthesis pathway and expression profiles of key structural genes in wheat roots under heat stress. (**A**) The proposed biosynthetic pathway of flavonoids in wheat. Red text indicates the key enzymes involved in each catalytic step. Dashed arrows represent multiple enzymatic steps or uncharacterized reactions. (**B**) Heatmap illustrating the expression patterns of candidate genes encoding the key enzymes shown in (**A**). Rows represent individual gene transcripts (labeled with gene IDs), and columns represent the different treatment groups for YM15 and YM158. The color scale indicates the relative expression levels, with red representing up-regulation and blue representing down-regulation. Abbreviations: CHS, Chalcone synthase; CHI, Chalcone isomerase; F3H, Flavanone 3-hydroxylase; F3′H, Flavonoid 3′-hydroxylase; F3′5′H, Flavonoid 3′,5′-hydroxylase; DFR, Dihydroflavonol 4-reductase; ANS, Anthocyanidin synthase.

KEGG enrichment analysis at the transcriptome level identified flavonoid biosynthesis as a crucial pathway activated in the heat-tolerant cultivar YM158 under high-temperature conditions. At the metabolome level, machine learning screening pinpointed naringenin, a key product of this pathway, as the primary biomarker for distinguishing heat tolerance differences. WGCNA further validated that the module significantly associated with heat tolerance was rich in structural genes involved in flavonoid biosynthesis. To explicitly establish the quantitative link between the transcriptome and metabolome, it is important to note that the dark-green module—which harbors these key structural genes—demonstrated a strong and significant quantitative correlation (r = −0.63, *p* = 0.005) with the accumulation of naringenin (Figure 6). Furthermore, the coordinated up-regulation of structural genes such as CHS and CHI (Figure 7) temporally and quantitatively parallels the substantial accumulation of naringenin and other downstream flavonoid metabolites detected in the metabolomic profile of the heat-tolerant cultivar YM158. Since analyses from these three dimensions converged on the same pathway, we confirmed that flavonoid biosynthesis is the core defense mechanism for YM158 against high-temperature stress. Consequently, we mapped the metabolic profile of this pathway and thoroughly analyzed the expression patterns of key enzyme genes using heat maps (Figure 7).

Chalcone synthase (CHS) serves as the initial rate-limiting enzyme in flavonoid synthesis. The heatmap reveals that several *CHS* family members, such as *LOC123055015* and *LOC123133080*, in YM158 display significant temperature-induced characteristics. Notably, at 30 °C, *CHS* expression in YM158 peaks, markedly surpassing that of YM15 during the same period. This suggests that YM158 can swiftly respond to heat signals, initiating flavonoid skeleton synthesis. Chalcone isomerase (*CHI*) converts chalcones into naringenin. In YM158, *CHI* genes, including *LOC123129897*, maintain high transcription levels under high-temperature stress, aligning with the substantial naringenin accumulation observed in the metabolome. Conversely, in YM15, this gene’s expression is significantly inhibited, hindering effective synthesis of downstream antioxidant substances. Additionally, downstream genes like flavonoid 3′-monooxygenase (*F3′H*) and flavonol synthase (*FLS*) exhibit notably high expression in YM158. The activation of these enzymes promotes the production of diverse flavonoid derivatives, such as luteolin and quercetin, further strengthening the chemical antioxidant barrier of the roots.

### 2.6. Validation by Quantitative Real-Time PCR

**Figure 8:**
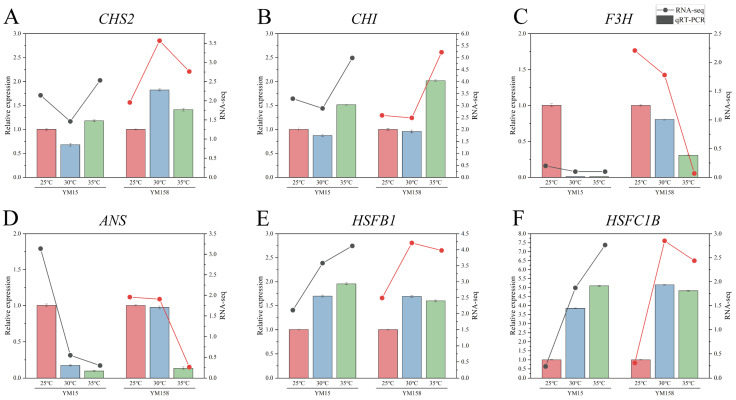
Validation of RNA-seq expression profiles by quantitative real-time PCR (qRT-PCR) for six key candidate genes. (**A**–**F**) Comparison of expression patterns for genes in YM15 and YM158 under 25 °C, 30 °C, and 35 °C treatments. (**A**) *CHS2*, LOC123047221, (**B**) *CHI*, LOC123105650, (**C**) *F3H*, LOC123042899, (**D**) *ANS*, LOC123140655, (**E**) *HSFB1*, LOC123103963, (**F**) *HSFC1B*, LOC123074721. The colored bars (left *y*-axis) represent the relative expression levels determined by qRT-PCR (n = 3), and the line graphs (right *y*-axis) represent the transcript abundance obtained from RNA-seq data. Error bars indicate standard deviation (SD). The consistencies between the qRT-PCR and RNA-seq trends confirm the reliability of the transcriptome data. The black line represents the RNA-seq results of YM15, and the red line represents the RNA-seq results of YM158.

To ensure the reliability of transcriptome sequencing (RNA-seq) data and confirm the expression patterns of key hub genes identified from our network analysis and crucial candidate genes under high-temperature stress, we conducted qRT-PCR analysis on six representative genes: *CHS2*, *CHI*, *F3H*, *ANS*, *HSF B-1*, and *HSF C-1b*. These genes were chosen based on their functional significance and positions within the co-expression network. The qRT-PCR results demonstrated that the expression trends of these six genes in both the heat-sensitive cultivar YM15 and the heat-tolerant cultivar YM158, across various temperature treatments, were highly consistent with the RNA-seq data (Figure 8).

## 3. Discussion

**Figure 9:**
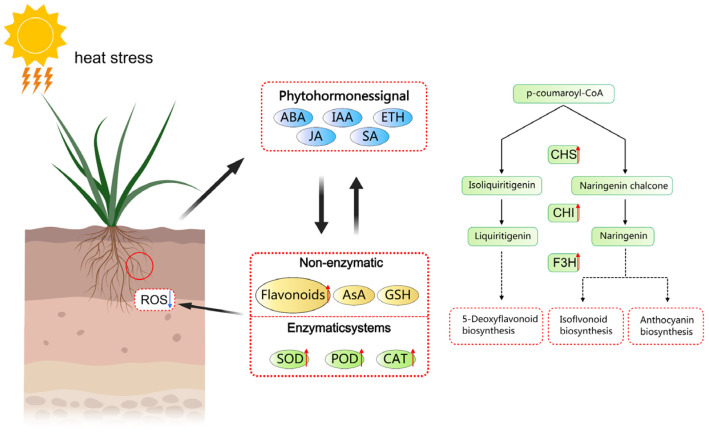
A proposed working model illustrating the regulatory network of flavonoid-mediated heat tolerance in wheat roots. Under heat stress, signal transduction pathways involving phytohormones (e.g., ABA, IAA, ETH) are activated. These signals coordinate the transcriptional upregulation of key structural genes (CHS, CHI, F3H) in the phenylpropanoid pathway, promoting the biosynthesis of specific flavonoids. The accumulated flavonoids serve as potent non-enzymatic antioxidants and work synergistically with the enzymatic antioxidant system (SOD, POD, CAT) to efficiently scavenge excess reactive oxygen species (ROS). This restoration of redox homeostasis protects cellular integrity and maintains root growth under elevated temperatures. Red upward arrows indicate upregulation of gene expression, increased enzyme activity, or metabolite accumulation. The blue downward arrow indicates the reduction in ROS levels. Solid arrows represent direct regulation or metabolic steps, while dashed arrows represent multi-step or indirect processes. The red circle indicates that the study focuses on wheat roots.

Extreme high-temperature events, driven by global warming, have become a major constraint on wheat yield [28]. Roots, as the primary organ for sensing soil environment changes, play a crucial role in determining a plant’s adaptability to high temperatures, which directly affects survival and productivity [29]. In this study, we conducted a comprehensive analysis of the response differences between the heat-tolerant wheat cultivar YM158 and the heat-sensitive cultivar YM15 under high-temperature stress. This was achieved by integrating phenomics, transcriptomics, metabolomics, and machine learning algorithms. Our core findings reveal a multi-dimensional heat-tolerance regulatory network, consisting of enzymatic and non-enzymatic antioxidant defense lines, protein homeostasis maintenance, and interactions between hormone and secondary-metabolite signals. Based on these findings, we proposed a putative working model to visually summarize these complex regulatory mechanisms (Figure 9).

High-temperature stress primarily causes cytotoxicity through oxidative damage from reactive oxygen species (ROS) bursts [30]. In this study, the heat-tolerant wheat cultivar YM158 demonstrated lower H_2_O_2_ accumulation and malondialdehyde (MDA) content compared to the heat-sensitive cultivar YM15 when exposed to extreme temperatures of 35 °C, while also maintaining higher root activity. This physiological resilience is evident in the plant’s morphogenesis. Although extreme heat inhibited the elongation of wheat’s main root, YM158 exhibited strong growth maintenance capabilities. Under high-temperature stress, YM158 maintained significantly greater root length and plant height than YM15. This phenotypic trait aligns with observations in other gramineous crops. Research on rice and maize shows that heat-tolerant germplasms often employ a growth maintenance strategy by preserving the activity and elongation capability of root tip meristem cells, thereby mitigating the inhibitory effects of high temperatures on primary root length and plant height [31,32]. This suggests that heat-tolerant cultivars can effectively counteract high-temperature inhibition of meristem cell division and elongation by maintaining intracellular redox homeostasis, resulting in superior growth compared to heat-sensitive cultivars [33].

Plants generally utilize two primary defense mechanisms to combat oxidative damage and protein denaturation resulting from reactive oxygen species (ROS) bursts: an enzymatic system led by antioxidant enzymes and a non-enzymatic system dominated by secondary metabolites [34,35]. Our research suggests that the heat tolerance of YM158 relies not on a single mechanism but on the synergistic enhancement of both systems.

At the enzymatic level, YM158, like heat-tolerant maize, sustains exceptionally high activities of SOD, POD, and CAT [36]. The dark-green module in the WGCNA corroborates this finding at the transcriptional level. GO enrichment results prominently highlight peroxidase activity, while PPI network analysis identifies the core hub gene of Cationic peroxidase *SPC4*. As a crucial component of the antioxidant enzyme system, *SPC4* high expression allows YM158 to efficiently catalyze the breakdown of excessive intracellular H_2_O_2_ [37]. This strategy of maintaining redox homeostasis through an efficient antioxidant enzyme system is highly conserved among gramineous crops. Previous research indicates that heat-tolerant rice and maize cultivars significantly upregulate similar POD and CAT activities under heat stress to prevent irreversible damage from membrane lipid peroxidation [38,39]. Additionally, some peroxidase family members possess auxin oxidase activity, directly participating in the oxidative degradation of auxin [40]. Consequently, the high expression of *SPC4* risks degrading auxin while scavenging ROS. However, YM158 accumulates auxin-binding substances (IAA-Glu) while sustaining high enzyme activity, suggesting a potential balance mechanism for auxin activity.

While the enzymatic system is essential, enzyme proteins are susceptible to inactivation at extremely high temperatures [41]. At this point, flavonoids, as potent non-enzymatic antioxidants, become particularly important [42]. The multi-omics evidence from this study highlights the central role of the flavonoid biosynthesis pathway in wheat’s heat tolerance. Notably, beyond the accumulation of common flavonoids like naringenin, we observed a specific activation of an atypical and often overlooked 5-deoxyflavonoid biosynthesis pathway in the roots of heat-tolerant wheat [43]. We detected a significant accumulation of key metabolites such as butin, garbanzol, and dihydrofisetin. Compared to regular 5-hydroxyflavonoids, these compounds exhibit greater hydrophobicity (lipophilicity) and unique metal-chelating properties [44]. This aligns with Brunetti et al.’s view that plants preferentially synthesize flavonoids with an ortho-dihydroxy B-ring, which possess high antioxidant capacity, to address intracellular redox imbalances under strong light or stress conditions [45]. We speculate that YM158 can incorporate these special lipophilic antioxidants into the phospholipid bilayer of the cell membrane, directly scavenging free radicals within the membrane and maintaining its physical stability.

Understanding how plants detect high-temperature signals and translate them into intracellular responses is a fundamental concern in stress biology [46]. Our WGCNA models identified several key regulatory nodes. Notably, the light-green module is related to the high-temperature response, a condition that physiologically causes iron overload. While the receptor kinase FER was identified in our initial network analyses, it should be noted that FER is upregulated at low temperatures; thus, its involvement in high-temperature adaptation likely represents a potential, indirect regulatory mechanism rather than a direct sensing role. We propose that these putative interactions between hormone signaling and secondary metabolism provide a hypothetical working model that warrants further experimental validation. FER plays a crucial role in monitoring plant cell wall integrity (CWI) [47]. We propose that YM158 utilizes FER to sense alterations in cell wall rheology induced by high temperatures, subsequently activating the downstream hormone signaling network. This process forms a tight feedback loop with flavonoid synthesis. Within the light-yellow module, we noted significant enrichment of the ABA, ethylene, and brassinosteroid (BR) signaling pathways, suggesting a complex hormone interaction network.

In YM158, we noted a significant enrichment of the abscisic acid (ABA) signaling pathway. Previous research has established that ABA is a key positive regulator of flavonoid synthesis [48]. It can markedly upregulate flavonoid structural genes by activating bZIP transcription factors and the MYB-bHLH-WD40 (MBW) transcription complex [49,50]. Concurrently, flavonoids play a vital role in modulating ABA signaling. Some studies suggest that flavonoids fine-tune ABA-induced stomatal closure and stress signal transduction by scavenging reactive oxygen species (ROS), thereby preventing cell death from excessive oxidative damage signals [51]. This feedback regulation mechanism between ABA-induced synthesis and flavonoids may be crucial for YM158 to maintain water balance and ROS homeostasis under high temperatures. We also observed significant enrichment of the ethylene signaling pathway in YM158. Ethylene pathway activation is not only a stress response but also accelerates flavonoid synthesis [52]. Research has shown that ethylene can directly bind to the MYB promoter through ethylene response factors (ERFs), and interact with JAZ proteins in the jasmonic acid (JA) signaling pathway to relieve the inhibition of the MYB complex, thereby synergistically promoting flavonoid biosynthesis [53,54,55]. This hormonal synergy ensures that YM158 can rapidly initiate secondary metabolic defenses during early high-temperature stress. Additionally, flavonoids inhibit polar auxin transport by blocking the activity of the serine-threonine kinase PINOID (PID) and altering the localization of auxin efflux proteins (PINs) on the cell membrane and endoplasmic reticulum [56]. They also inhibit the activity of auxin oxidase DAO1, reducing auxin degradation [57]. Coupled with the accumulation of IAA-Glu (conjugated auxin) observed in YM158, we speculate that under high temperatures, heat-tolerant cultivars synthesize large amounts of flavonoids. These flavonoids locally inhibit auxin efflux at the root tip and reduce degradation by inhibiting DAO1, thus maintaining high auxin concentrations in the root tip meristem. This high-auxin environment is crucial for sustaining stem cell activity and root meristematic ability, preventing meristem exhaustion due to high temperatures [58]. Interestingly, previous studies have noted that high auxin concentrations, in turn, inhibit flavonoid synthesis, indicating a precise negative feedback homeostasis mechanism in YM158 to prevent excessive energy consumption in the defense response.

The deeper mechanism of heat tolerance involves maintaining protein homeostasis. Our transcriptome KEGG enrichment analysis identified a critical spatio-temporal distinction: at the lethal temperature of 35 °C, YM158 specifically activated the endoplasmic reticulum protein processing pathway, whereas YM15 did not effectively initiate this mechanism. High temperatures can cause nascent peptide chains to misfold or mature proteins to denature, leading to endoplasmic reticulum stress (ER Stress) [59]. In YM158, the rapid upregulation of heat shock factors, including the hub genes *HSF B-1* and *HSF C-1b*, facilitated the synthesis of heat shock proteins (HSPs) and molecular chaperones. These chaperone proteins assist in refolding denatured proteins or degrading misfolded proteins via the ERAD pathway [60]. Conversely, although YM15 attempted to sustain basic metabolism, such as photosynthesis-related genes, under high temperatures, the absence of an effective protein repair mechanism ultimately resulted in cellular function collapse. This finding underscores the unfolded protein response (UPR) as crucial for the survival of wheat roots under extreme high temperatures.

In traditional omics analysis, researchers often struggle to differentiate between passenger differences and driver differences when dealing with tens of thousands of genes and metabolites [25]. This study introduces machine learning algorithms, such as random forest and SVM, to successfully identify 9 key biomarkers from 3546 metabolic features. These biomarkers not only contribute significantly to classification but also hold clear biological significance (e.g., IAA-Glu and naringenin). Machine learning addresses the limitations of traditional differential analysis, which focuses solely on fold change, by identifying features with small fold changes that play central roles in regulatory networks [61]. This approach enhances our understanding of the secondary metabolic profile of wheat roots and highlights the potential of data-driven machine learning strategies in unraveling the mechanisms underlying complex agronomic traits.

While our multi-omics integration provides valuable mechanistic insights into wheat root thermotolerance, we acknowledge certain limitations in the current study. Although the expression trends of key hub genes were validated via qRT-PCR, direct functional validation—such as through CRISPR/Cas9-mediated gene editing or overexpression transgenic lines—remains to be conducted in future studies to definitively confirm their causal roles. Despite this limitation, our findings hold significant practical implications for agricultural improvement. The key metabolic biomarkers identified here, particularly specific flavonoids like naringenin, have the potential to serve as effective metabolic markers for the rapid screening and evaluation of heat-tolerant wheat germplasms in breeding programs. Furthermore, the core hub genes mapped within the flavonoid biosynthesis and hormone signaling networks offer promising genetic targets for precise molecular breeding or genetic engineering strategies, ultimately facilitating the development of climate-resilient crop varieties.

## 4. Materials and Methods

### 4.1. Plant Materials and Temperature Treatments

In this study, wheat cultivars Yangmai 15 and Yangmai 158, sourced from the Lixiahe Agricultural Science Research Institute (Yangzhou, China), were used. The wheat was hydroponically cultivated in a controlled environment chamber with Hoagland nutrient solution (Table S1) for 5 days. Afterward, the seedlings were subjected to temperature gradient treatments at 25 °C, 30 °C, and 35 °C. The photoperiod was maintained at 16 h of light and 8 h of darkness, with humidity at 75% RH and light intensity at 10,000 LUX.

### 4.2. Evaluation of Agronomic Traits

Seedling growth was continuously monitored, with samples collected on the 5th day of treatment. For each treatment group, 100 seedlings (n = 20) were selected. Morphological imaging of wheat roots was performed using the MICROTEK ScanMaker i850 root scanner (Shanghai, China) and plant root analysis instrument system (Version: LS—A, Hangzhou Wanshen Detection Technology Co., Ltd., Hangzhou, China), measuring root length and the number of lateral roots. Each parameter for each seedling was measured in three technical replicates. Statistical analysis was conducted on the average values from 100 independent seedlings in each treatment group (n = 100).

### 4.3. Physiological Measurements

Following the instructions provided by the ELISA kit from Shanghai Qiaoling Biotechnology Co., Ltd. (Shanghai, China), wheat root tissue samples were carefully collected, rinsed with PBS at 4 °C, and dried. Precisely 1 g of the samples was weighed, chopped into small pieces, and placed in a 10 mL homogenization tube. A homogenization medium was added at a weight-to-volume ratio of 1:9 (g:mL), and the samples were thoroughly ground in an ice-water bath to create a 10% homogenate. The homogenate was then centrifuged at approximately 3000 rpm for 15 min, after which the supernatant was collected to assess physiological indicators such as MDA, root activity, and H_2_O_2_. For the determination of antioxidant enzyme activities, the activity of superoxide dismutase (SOD) was measured using the nitroblue tetrazolium (NBT) method. Peroxidase (POD) activity was assessed through the guaiacol method, and catalase (CAT) activity was determined using ultraviolet spectrophotometry [62,63].

### 4.4. Metabolite Extraction and Metabolomics Analysis

Metabolome analysis was carried out by Shanghai OE Biotech Co., Ltd. In summary, freeze-dried leaf samples were processed using a mixer mill (MM 400, Retsch Technology, Haan, Germany). Two small steel balls and 600 μL of methanol-water (*v*/*v* = 7:3) containing a mixed internal standard (4 μg/mL) were added. Metabolite extraction was performed on leaves from three treatment groups, with six biological replicates each to ensure statistical reliability. Samples were pre-cooled at −40 °C for 2 min before being ground in the mill at 60 Hz for 2 min. They underwent ultrasonic extraction in an ice-water bath for 30 min, followed by incubation at −40 °C for 2 h. The samples were then centrifuged at 13,000 rpm for 20 min at 4 °C. A syringe was used to carefully collect 150 μL of the supernatant, which was transferred to an LC injection vial. The samples were stored at –80 °C until analyzed by LC–MS. The analysis utilized a liquid chromatography-mass spectrometry system, comprising a Waters ACQUITY UPLC I-Class Plus (Milford, USA) and a Thermo Q Exactive high-resolution tandem mass spectrometer (Waltham, USA) [64].

Leaves from the three treatment groups were used for RNA extraction, with all RNA-Seq experiments conducted in triplicate to ensure robust statistical analysis. Total RNA was extracted using TRIzol reagent, adhering to the manufacturer’s protocol. The RNA’s purity and concentration were measured with a NanoDrop 2000 spectrophotometer (Thermo Scientific, Waltham, MA, USA), while its integrity was assessed using an Agilent 2100 bioanalyzer (Agilent Technologies, Santa Clara, CA, USA). Transcriptome libraries were prepared using the Vazyme’s VAHTS Universal V6 RNA-Seq Library Prep Kit (Nanjing, China), following the manufacturer’s instructions. Transcriptome sequencing and subsequent data analysis were carried out by Shanghai OE Biotech Co., Ltd. (Shanghai, China) on the Illumina platform [65].

The data matrix was imported into the R package for principal component analysis (PCA) to assess the overall sample distribution and ensure analysis stability. To differentiate metabolites among groups, orthogonal partial least squares discriminant analysis (OPLS-DA) and partial least squares discriminant analysis (PLS-DA) were employed. To avoid overfitting, 7-fold cross-validation and 200 response permutation tests (RPT) were conducted to evaluate model quality. The variable importance in projection (VIP) values from the OPLS-DA model ranked each variable’s contribution to group discrimination. A two-tailed Student’s *t*-test was used to determine the significance of differences in genes/metabolites between groups. Differential metabolites with VIP values greater than 1.0 and *p*-values less than 0.05 were selected.

### 4.5. Machine Learning Algorithms and Weighted Gene Co-Expression Network Analysis

This study utilized a Random Forest (RF)-based feature selection algorithm to identify key metabolites associated with heat tolerance from high-dimensional metabolomic data and to differentiate the metabolic strategies of two cultivars, YM15 and YM158, under high-temperature stress.

A classification model was developed using the randomForest package (v4.7-1) in the R programming environment. The target variable was temperature treatment (25 °C, 30 °C, 35 °C), while all metabolic features served as predictor variables. To isolate genotype-specific response patterns, random forest classifiers were independently constructed for the two cultivars, YM15 and YM158. Each model was configured with 1000 decision trees, and the number of candidate variables for node splitting was set to the square root of the total number of variables to ensure model robustness [66].

The Mean Decrease Accuracy (MDA) was used as the primary indicator to assess the significance of metabolites. A higher MDA value signifies that the metabolite is more critical in differentiating between various temperature states. To evaluate the similarities and differences in the metabolic response strategies of the two cultivars, we performed a Spearman rank correlation analysis on the feature importance rankings of YM15 and YM158.

The gene/metabolite co-expression network was developed utilizing the WGCNA package in R version 4.0.0, with parameters set as follows: Power = 9, MinModuleSize = 50, MergeCutHeight = 0.25, and TOMType = unsigned. To define and identify hub genes within the critical modules, we utilized the degree centrality metric from the constructed Protein–Protein Interaction (PPI) networks. Specifically, genes that exhibited the highest intramodular connectivity and ranked within the Top 50 nodes based on the number of interacting edges (degree) were designated as core hub genes.

### 4.6. Quantitative Real-Time PCR Validation

cDNA synthesis was performed with total RNA and HiScript II Q RT SuperMix (Vazyme). Primers, designed using Oligo 7.0 (Table S2), were employed for qRT-PCR amplification alongside SYBR Master Mix (Vazyme). Relative expression levels were determined using the 2^−ΔΔCt^ method (n = 3) [67]. Statistical analysis of the data was conducted using the LSD test, with significance set at *p* < 0.05.

### 4.7. Statistical Analysis

We conducted statistical analyses using SPSS 25.0 software (IBM, Armonk, NY, USA), applying Duncan’s Multiple Range Test (MRT) to determine statistical significance at *p* < 0.05. Graphical representations were created with GraphPad Prism 8.0, and data are expressed as mean ± S.D.

## 5. Conclusions

This study reveals that when subjected to varying levels of heat stress, heat-tolerant wheat cultivars undergo multiple complex molecular and metabolic adaptations in their roots to shield cells from oxidative stress, heat damage, and protein denaturation. These adaptations involve potential perception and remodeling of cell wall integrity, maintenance of protein homeostasis via the endoplasmic reticulum protein processing system, specific activation of the flavonoid biosynthesis pathway, and coordinated enhancement of the antioxidant enzyme system. Additionally, changes in the expression of genes encoding heat-shock transcription factors, cell wall receptor kinases, and hormone signal transduction elements (particularly abscisic acid, ethylene, and auxin) are crucial. While these changes collectively enhance the heat tolerance of YM158 in high-temperature environments, the complex interaction between hormone signaling and secondary metabolism may offer ideal targets for deepening our understanding of this species’ heat adaptation mechanisms and potentially improving wheat’s heat-tolerant traits through molecular breeding strategies.

## Data Availability

NCBI Sequence Read Archive (SRA): BioProject ID: PRJNA1420966.

## Supplementary Materials

The following supporting information can be downloaded at: https://www.mdpi.com/article/10.3390/plants15060965/s1, Table S1. Nutrient elements and their amounts (ppm) used in Hoagland nutrient solution. Table S2. Primer information for RT-qPCR analysis. Table S3. Protein-Protein Interaction network of Darkgreen. Table S4. Protein-Protein Interaction network of Lightyellow.
