# Supplementary material for: Integrated Transcriptomics and Metabolomics with Machine Learning Identify Flavonoids as Key Effectors in Wheat Root Thermotolerance
Source: Plants (Basel). 2026 Mar 20;15(6):965. doi: 10.3390/plants15060965 (PMC13030383; doi:10.3390/plants15060965)
Supplement: Supplementary file 1 [file plants-15-00965-s001.zip › plants-4176087-supplementary.pdf]

Supplementary Table S1 Nutrient elements and their amounts (ppm) used in Hoagland nutrient solution

Elements	(ppm)
Nitrogen (N)	210
Phosphorus (P)	30
Potassium (K)	234
Magnesium (Mg)	48
Calcium (Ca)	200
Sulfur (S)	64
Iron (Fe)	5
Manganese (Mn)	0.5
Bor (B)	0.5
Copper (Cu)	0.02
Zinc (Zn)	0.05

Supplementary Table S2 Primer information for RT-qPCR analysis

Gene ID	Gene name	Forward Primer	Reverse Primer
LOC123097805	TaEFα	5' GGTTAAGATGATTCCCACCAAGCC 3'	5' GACAACACCAACAGCAACAGTCTG 3'
LOC123047221	CHS2	5' GGAGAAGTTCAAGAGGATGTGTGA 3'	5' GGCGCAAGCCGAGCAT 3'
LOC123105650	CHI	5' GGAGATCGGCGGCAACTT 3'	5' AACTTCTCGAACTCGCCGGT 3'
LOC123042899	F3H	5' CGACCACGGCCATTACTTGA 3'	5' GAGGTCGCCCTCCATCTTG 3'
LOC123140655	ANS	5' TCACCAAGACCTTCGGGGAG 3'	5' AGGTCGTCCTCCACCTGTGT 3'
LOC123103963	HSFB1	5' CAGCAGCAGAAGGGTGGTG 3'	5' ACCTTCCGAAAACCGTAGGTG 3'
LOC123074721	HSFC1B	5' GCCAAGACGTTCCACATGGT 3'	5' CCTTGCGAAAACCGTAGGTG 3'

Supplementary Table S3 Protein-Protein Interaction network of Darkgreen

Node name	Protein name
SPC4	Cationic peroxidase SPC4
DTX34	Protein DETOXIFICATION 34
ANS2	Leucoanthocyanidin dioxygenase 2
CYP75B3	Flavonoid 3'-monooxygenase CYP75B3
F3H	Naringenin,2-oxoglutarate 3-dioxygenase
CHS2	Chalcone synthase 2
GRXC2	Putative glutaredoxin-C2
ACD6.1	Protein ACCELERATED CELL DEATH 6
ACD6.2	Protein ACCELERATED CELL DEATH 6
CLP.1	Chitinase CLP
CLP.2	Chitinase CLP
RGA2	Disease resistance protein RGA2
AGP25	Classical arabinogalactan protein 25
RGA1	Putative disease resistance protein RGA1
CIN4	Beta-fructofuranosidase, insoluble isoenzyme 4
LSI2	Silicon efflux transporter LSI2
SAG12.1	Senescence-specific cysteine protease SAG12
SAG12.2	Senescence-specific cysteine protease SAG12
BGLU7	Beta-glucosidase 7
UGT73C3	UDP-glycosyltransferase 73C3
CYP72A616	Cytochrome P450 CYP72A616
CRK6.1	Cysteine-rich receptor-like protein kinase 6
CRK6.2	Cysteine-rich receptor-like protein kinase 6
Xyl2	Beta-xylosidase/alpha-L-arabinofuranosidase 2
CYP51	Obtusifoliol 14-alpha demethylase
H4.1	Histone H4 variant TH011
BURP11	BURP domain-containing protein 11
0	Histone H4 variant TH011
FMO1	Probable flavin-containing monooxygenase 1
H4.2	Histone H4 variant TH011
Serpin-like	Putative serpin-Z12
H2A	Probable histone H2A.5
WAK1	Wall-associated receptor kinase 1
EAP	Aspartyl protease family protein At5g10770
H4.3	Histone H4 variant TH011
H2B.3	Histone H2B.3
H4.4	Histone H4 variant TH011
MIK2	MDIS1-interacting receptor like kinase 2
CYP89A2	Cytochrome P450 89A2
GOS9	Protein GOS9
ervatamin-B	Ervatamin-B OS=Tabernaemontana divaricata

Supplementary Table S4 Protein-Protein Interaction network of Lightyellow

Node name	Protein name
GAP1	GTPase activating protein 1
WNK5	Probable serine/threonine-protein kinase WNK5
FER	Receptor-like protein kinase FERONIA
PBL7	Probable serine/threonine-protein kinase PBL7
PP2C	Probable protein phosphatase 2C 32
HSFC1B	Heat stress transcription factor C-1b
NAC074	NAC domain-containing protein 74
PUB16	U-box domain-containing protein 16
NET4A	Protein NETWORKED 4A
PSI3	Protein PSK SIMULATOR 3
HSFC1B	Heat stress transcription factor C-1b
WRKY6	WRKY transcription factor 6
NAC074	NAC domain-containing protein 74
CYP450	Cytochrome P450 CYP72A616
PER56	Peroxidase 56
NHL10	NDR1/HIN1-like protein 10
ACR8	ACT domain-containing protein ACR8
ASP1	Aspartic proteinase Asp1
HERK1	Receptor-like protein kinase HERK 1
CCX1	Cation/calcium exchanger 1
GLC1	Glucan endo-1,3-beta-glucosidase
ASP1	Aspartic proteinase Asp1
TSPO	Translocator protein homolog
B3GALT7	Beta-1,3-galactosyltransferase 7
HSFB1	Heat stress transcription factor B-1
WRKY1	Protein WRKY1
LURP15.1	Protein LURP-one-related 15
HVA22	Protein HVA22
PBL19	Probable serine/threonine-protein kinase PBL19
PBL19	Probable serine/threonine-protein kinase PBL19
LURP15.2	Protein LURP-one-related 15
MDIS2	Protein MALE DISCOVERER 2
BAM1	Leucine-rich repeat receptor-like serine/threonine-protein kinase BAM1
KCS11	3-ketoacyl-CoA synthase 11
ERD7	Protein EARLY-RESPONSIVE TO DEHYDRATION 7, chloroplastic
SLAH2	S-type anion channel SLAH2
GAP1	GTPase activating protein 1
